# Group size predicts prenatal investment and offspring growth in a cooperatively breeding bird

**DOI:** 10.1093/beheco/arag095

**Published:** 2026-09-03

**Authors:** Andries K Janse van Vuuren, Roberto Vento, Ara Monadjem, Kat Bebbington, Kevin D Matson, Sjouke A Kingma

**Affiliations:** Behavioural Ecology Group, Department of Animal Sciences, Wageningen University and Research, P.O. Box 338, 6700 AH, Wageningen, The Netherlands; Behavioural Ecology Group, Department of Animal Sciences, Wageningen University and Research, P.O. Box 338, 6700 AH, Wageningen, The Netherlands; Behavioural and Physiological Ecology, Groningen Institute for Evolutionary Life Sciences, University of Groningen, P.O. Box 11103, 9700 CC Groningen, Netherlands; Mammal Research Institute, Department of Zoology and Entomology, University of Pretoria, Private Bag X20, Hatfield 0028, Pretoria, South Africa; Department of Biological Sciences, University of Eswatini, Private Bag 4, Kwaluseni M201, Eswatini; Behavioural Ecology Group, Department of Animal Sciences, Wageningen University and Research, P.O. Box 338, 6700 AH, Wageningen, The Netherlands; Wildlife Ecology and Conservation Group, Department of Environmental Sciences, Wageningen University & Research, P.O. Box 47, 6700 AA Wageningen, The Netherlands; Wildlife Ecology and Conservation Group, Department of Environmental Sciences, Wageningen University & Research, P.O. Box 47, 6700 AA Wageningen, The Netherlands; Behavioural Ecology Group, Department of Animal Sciences, Wageningen University and Research, P.O. Box 338, 6700 AH, Wageningen, The Netherlands

**Keywords:** cooperative breeding, maternal investment, load lightening, egg size, offspring condition, compensatory growth

## Abstract

Cooperative breeding, where seemingly altruistic helpers forgo reproduction to assist in raising the offspring of others, is a puzzle for biologists because it appears to contradict evolutionary principles of selfishness. One potential explanation is that helpers enhance group productivity, thus improving the direct fitness of breeders and providing indirect genetic and group-living benefits for helpers. In many species, however, helper presence does not actually enhance offspring production, raising the question of whether helpers instead either improve offspring quality (rather than quantity) or allow breeders to reduce prenatal investment (thereby increasing breeders’ condition and future reproduction). In arrow-marked babblers (*Turdoides jardineii*), helpers do not increase the number of fledglings produced. To test whether helpers instead improve offspring quality or compensate for reduced prenatal investment, we investigated how egg volume, offspring growth, and nestling provisioning rates vary with group size. Breeding females in larger groups laid smaller eggs, but also produced heavier fledglings than those from smaller groups. This suggests that helpers in larger groups not only facilitate reduced prenatal maternal investment, but actually over-compensate for reduced egg sizes. Interestingly, larger groups did not feed nestlings more, suggesting that over-compensatory nestling growth may result from larger groups providing higher-quality food. Our results show that helping can be selected for via different mechanisms acting on both breeders and offspring, and that these can apply simultaneously within a single species. We highlight that variation in life history may explain inter-specific differences in mechanisms by which helpers improve group productivity.

## Teaser

In arrow-marked babbler groups, helpers (non-breeding birds who forego their own reproduction to help raise young of the breeding pair instead) enhance the growth of offspring of the breeding pair. This seems to enable breeding females to lay smaller eggs because helpers compensate for the initially smaller offspring. Thus, cooperative breeding in this species is under selection through the dual benefits of reduced workload of the breeding female and increased offspring growth rate. This contributes to our understanding of the evolutionary benefits of cooperative breeding -- something that has long puzzled evolutionary biologists because the evolutionary benefits are not always clear.

## Introduction

Cooperative breeding, in which some individuals (“helpers”) delay or forego their own reproduction to assist others (“breeders”) in rearing offspring, occurs in birds (∼9% of species), mammals (3%), and some fish (Solomon and French 1997; Cockburn 1998; Koenig and Dickinson 2004, 2016). This breeding system has received considerable attention as it offers important insights into the evolution of sociality and cooperative behavior (Solomon and French 1997; Cockburn 1998; Koenig and Dickinson 2004, 2016). Most notably, living and breeding in such cooperatively breeding groups is a puzzle for evolutionary biologists because from the breeder's perspective, sharing resources (and sometimes even reproduction) with helpers may reduce their own direct fitness, whereas from the helper's perspective, it is costly to raise offspring of others and to forgo one's own reproduction (Koenig and Dickinson 2004, 2016). How such systems can remain stable is therefore not immediately clear from an evolutionary perspective, which assumes individuals to act in ways which improve their own fitness.

One potential answer to why cooperative breeding evolves or persists is that the presence of helpers may enhance group productivity: if so, producing more offspring improves direct fitness of breeders, whereas providing helpers with other fitness benefits such as increased survival in resulting larger groups or indirect (kin-selected) benefits (Kingma et al. 2014; West et al. 2015; Shen et al. 2017). In several cooperatively breeding species, helpers indeed directly improve the reproductive success of the group, eg because the additional care they provide reduces offspring starvation or predation (reviewed in Hatchwell 1999; Kingma et al. 2010; Downing et al. 2020). However, helpers do not improve reproductive success in all species (see Hatchwell 1999), and in such cases, other, perhaps more indirect, mechanisms by which helpers improve group productivity must play a larger role. One way in which help in offspring care can enhance reproductive output is through positive effects on offspring condition or quality, thereby improving the performance of such offspring in the future (Clutton-Brock 2002; Hatchwell et al. 2004). Interestingly, however, in some species, neither offspring number nor quality appears to be affected by helpers (eg Russell et al. 2007).

How selection acts on helping behavior is not exclusively determined by helper effects on current offspring, but varies according to the relative contribution which future reproduction makes to lifetime reproductive success. As such, helper contribution may improve future reproductive success if it lightens the current prenatal or postnatal workload of breeders, allowing breeders to conserve energy for their own survival and future reproduction (“load-lightening hypothesis”; Crick 1992; Hatchwell 1999; Russell et al. 2007). Work grounded in the load-lightening hypothesis has shown that breeders in some species reduce their prenatal investment when additional postnatal care by helpers leads to increased offspring growth rates and thus compensates for reduced initial offspring size (Raihani and Ridley 2007; Russell et al. 2007; Dixit et al. 2017). Importantly, prenatal load-lightening can make the reproductive benefits of helping difficult to detect if helpers fully compensate for the reduction in prenatal investment by breeders and the overall number and quality of offspring are therefore unrelated to helper presence (known as the “concealed-helper-effect” hypothesis; Russell et al. 2007; Koenig et al. 2009; Dixit et al. 2017).

In birds, egg production is energetically demanding (Monaghan and Nager 1997; Williams 2005; Gil 2008), and reduced resource allocation in eggs may allow for faster re-nesting, greater longevity, and/or increased future reproductive success (Russell et al. 2007). Such patterns of reduced investment align with the slow pace of life that is commonly associated with cooperative breeders, because these often inhabit harsh environments, often have long breeding seasons and are often relatively long-lived (Arnold and Owens 1998; Covas and Griesser 2007; Jetz and Rubenstein 2011; Griesser et al. 2017). Empirical tests of prenatal load-lightening—ie the idea that maternal investment is reduced when helpers are present—have produced mixed results across cooperatively breeding birds (reviewed by Dixit et al. 2017). In some species, such as superb fairy-wrens (*Malurus cyaneus*; Russell et al. 2007) and carrion crows (*Corvus corone*; Canestrari et al. 2011), females lay smaller eggs when assisted by (more) helpers, supporting the hypothesis. Other studies report no significant relationship between helper number and egg size, as in Tibetan ground tits (*Pseudopodoces humilis*; Zhao et al. 2019) and sociable weavers (*Philetairus socius*; Fortuna et al. 2021), whereas some studies have even reported the opposite pattern, with females in larger groups laying larger eggs (such as white-browed sparrow-weavers (*Plocepasser mahali*; Capilla-Lasheras et al. 2023) and placid greenbuls (*Phyllastrephus placidus*; Van de Loock et al. 2023)). Why the effects are so different across species remains unclear (Dixit et al. 2017) and warrants further research, especially because many additional factors, including the timing of breeding, habitat quality, food availability, and social factors, can affect maternal investment (Monaghan and Nager 1997; Mousseau and Fox 1998). Several explanations thus may account for this heterogeneity, based on differences in life history and ecology across species. The most beneficial level of maternal investment likely depends on the prioritization of offspring growth versus self-preservation (ie current vs future reproduction).

In species with high rates of nest predation, females may benefit from preserving energy for rapid re-nesting after a brood fails by laying smaller eggs when helpers are present. In contrast, in species where entire brood failure is rarer or breeding opportunities are limited, selection may favor maintaining or even increasing prenatal investment to promote nestling survival or development. To understand how life history and ecological context shape these contrasting patterns, more tests of prenatal load-lightening which simultaneously measure prenatal maternal investment and helper contribution, as well as their combined effect on offspring growth, are needed (Russell et al. 2007; Canestrari et al. 2011; Dixit et al. 2017; Van de Loock et al. 2023).

In this study, we test a set of different hypotheses related to the benefits of helping in cooperatively breeding arrow-marked babblers (*Turdoides jardineii*; hereafter, babbler), a year-round territorial, medium-sized passerine. Babblers are obligate cooperative breeders with variably sized groups: a breeding pair is assisted by 1 to 8 subordinate helpers of both sexes which contribute to territory defense, incubation, and nestling food provisioning (Monadjem et al. 1995; Hockey et al. 2005). Previous work in this species did not reveal an increase in fledging numbers with group size (Monadjem et al. 1995), raising the question of whether other benefits, ie increased offspring quality (rather than quantity) or breeder load-lightening, may drive cooperative breeding in this species. Babblers are long-lived, have a relatively long breeding season in a moderately unpredictable environment, and experience a high rate of nest failure due to predation (Hockey et al. 2005; manuscript in prep.). Breeder load-lightening, as a mechanism to facilitate fast renesting and future survival, may therefore be a particularly important benefit of helping in this species.

We first tested (and confirmed) that group size does not increase clutch size or fledgling number in our study population (as in Monadjem et al. 1995). Subsequently, we explored whether helpers instead provide subtler benefits by altering maternal investment and/or nestling development. To do so, we tested whether egg size, nestling growth, and nestling provisioning rates varied in relation to group size. If helper benefits are mainly related to improving offspring quality, we predicted that provisioning rates and offspring growth would be greater in larger groups, but that egg size would not vary with group size. If helper benefits mainly relate to load-lightening of breeders, we predicted that breeding females in larger groups would lay smaller eggs. If helper effects are concealed in babblers (under the concealed-helper-effect hypothesis), we predicted that helpers compensate fully for reduced egg size such that offspring condition does not vary with group size. Revealing whether or not group size relates to prenatal investment in egg size and offspring growth expands on our understanding of how cooperation can be maintained in this species and other cooperative breeders where helpers do not appear to directly enhance the number of fledglings.

## Materials and methods

### Study site

The study was conducted in Mbuluzi Game Reserve in Eswatini, southern Africa (26°8′ S, 32°0′ E). Our study area is characterized by dense riverine forests, deciduous thickets, and acacia savanna bushveld. The region experiences seasonal variation in temperature and rainfall, with usually dry, cool winters and hot, wet summers, although, within breeding seasons, climatic circumstances are also highly variable. Data were collected over 4 seasons (2019/2020, 2022/2023, 2023/2024, 2024/2025), from August to January, which represents the peak breeding season in our population. However, we cannot preclude that some nesting might have taken place after the end of our study. Adult babblers were caught in mist nets and ringed with a SAFRING numbered metal ring and a unique combination of 3 color-rings (under SAFRING licenses 17811 and 17812).

### Study species

The arrow-marked babbler is a medium-sized (23 to 25 cm, 56 to 85 g) obligate cooperative breeder inhabiting a range of savannah ecosystems across sub-Saharan Africa (Hockey et al. 2005). Group members feed together in leaf litter on insects and small vertebrates. Year-round stable groups consist of 3 to 10 individuals. Territories are stable across years (although boundaries can change), with groups maintaining strong year-round territoriality (Monadjem et al. 1995; Hockey et al. 2005; personal observation). Boundaries are actively defended (or shifted) through regular confrontations with neighboring groups along territory edges (Hockey et al. 2005; personal observation). In a closely related species (southern pied babbler; *Turdoides bicolor*), groups consist of a breeding male and female with multiple related and unrelated helpers (Nelson-Flower et al. 2011). Although this has not yet been directly confirmed in arrow-marked babblers, our surveys on the movement of ringed birds (who remained as subordinates in their natal group but sometimes moved to other, unrelated, groups) and our observations of small, constant clutch sizes (usually 2 to 4 eggs, indicating that only 1 female laid eggs) suggest that group composition is similar in our study species (Hockey et al. 2005). Nests are well-concealed cups constructed of thick grasses, which are newly constructed in different locations within the territory for each breeding attempt. Nestlings remain in the nest for ∼12 to 21 days before fledgling and remain dependent on the group for at least 2 months (Monadjem et al. 1995; Hockey et al. 2005; personal observation).

### Nest searching and monitoring

Because babbler group members move together as a cohesive unit (Hockey et al. 2005), we could confidently map territories (based on territorial interactions with neighboring groups, including vocal face-offs and physical confrontations at territorial borders; Monadjem et al. 1995) and quantify group size throughout each season. We identified groups and nest ownership based on their location and the presence of color-ringed individuals in the group.

To find nests, we performed systematic transect nest searches (walking ∼30 m between transect lines) across our study site (∼5 km^2^) at least weekly to ensure nests were found at an early stage. In total, we found 69 nests, of which 15 were in the building stage, 42 contained 1 or more eggs, and 12 contained nestlings. Nests were sometimes also found during observing the movements and behavior of individuals within groups.

Once a nest was found, a solar-powered motion-triggered camera (Wilsus Tradenda wireless, by Wildlife Monitoring Solutions) was placed ∼2 m from the nest to determine when the nest was active, when and how many nestlings fledged, and to detect predation or nest parasitism events. Nests were monitored physically every ∼4 days to determine egg laying and hatching dates, egg and clutch size, number of nestlings, and nestling survival, and to ensure cameras had enough battery power and SD card memory space.

### Egg measurements

We measured eggs using digital sliding calipers (width and length, to the nearest 0.1 mm), and eggs were marked with small dots using a nontoxic marker during the first measurement to allow identification of newly laid eggs. Egg volume was calculated using Hoyt's (1979) method:


$$V=0.51\times L\times W^{2}$$

where *L* is egg length (mm), *W* is egg width (mm), and *K* is a shape constant for passerines (Hoyt 1979).

The eggs of the Levaillant's cuckoo (*Clamator levaillantii*), which sometimes parasitize babbler nests, were discerned based on color (cuckoo eggs are slightly greener), size (cuckoo eggs are slightly wider), and number (ie if an additional egg appeared more than 2 days after initial clutch completion), and confirmed using video footage of parasitism events (cuckoo eggs: *n* = 24 eggs from 17 nests).

### Nestling measurements

Nestlings were ringed and measured on day 11 after hatching (hatch date = day 1) in the morning between 10:00 and 12:00 h. Hatch dates were determined using a combination of more frequent nest checks around the expected hatching period, changes in adult behavior at the nest observed from video footage, and age of nestlings based on their physical characteristics. Nestling body mass (±0.1 g) was measured using a Pesola spring scale. In addition, nestlings from the 2023/2024 and 2024/2025 breeding seasons were also weighed on day 6 (05:00 to 07:00 h) to allow estimations of nestling growth between day 6 and 11, and to assess whether nestlings in larger groups were initially smaller at an earlier growth stage (as predicted by the “concealed-helper-benefit” hypothesis; Russell et al. 2007). Because nestlings were too small to ring on day 6, we clipped a very small piece of a different toenail of each nestling, so we could recognize each nestling on day 11 and determine individual growth rate between day 6 and 11. We analyzed the relationship between group size and (i) nestling mass on day 6, (ii) nestling mass on day 11, and (iii) the growth rate from day 6 to day 11 (increase in g day^−1^) (see below). Some nestlings were measured 1 day around day 6 or 11 to avoid disturbing nests on rainy days (see Table 1). In general, we refer to only the intended measurement moments (ie mass at day 6 and day 11), but when relevant, we statistically correct for actual age (see below).

**Table 1:** The effect of group size on the body mass of (a) 6-day-old and (b) 11-day-old arrow-marked babbler nestlings, and (c) growth rate (mass increase) between these stages.

	(a) Nestling mass day 6 (*n* = 27 nestlings, 13 nests, 11 groups)			(b) Nestling mass day 11 (*n* = 51 nestlings, 24 nests, 17 groups)			(c) Nestling growth rate (*n* = 19 nestlings, 9 nests, 8 groups)		
	Estimate ± SE	*t*	*P*	Estimate ± SE	*t*	*P*	Estimate ± SE	*t*	*P*
Intercept	4.355 ± 13.495	…	…	43.520 ± 12.383	…	…	1.339 ± 0.850	…	…
Group size	−0.651 ± 0.489	−1.332	0.195	1.574 ± 0.443	3.553	**0**.**002**	0.284 ± 0.124	2.296	0.050
Age	4.256 ± 2.025	2.102	**0.046**	−0.972 ± 1.068	−0.911	0.374	…	…	…
**Random intercepts**	**Variance ± SD**			**Variance ± SD**			**Variance ± SSD**		
Nest identity	0.000 ± 0.000	…	…	4.069 ± 2.017	…	…	0.078 ± 0.280	…	…
Group identity	0.000 ± 0.000	…	…	1.859 ± 1.363	…	…	0.000 ± 0.000	…	…

Sample sizes are given as the number of nestlings and the number of nests. Because measurements were not always possible on day 6 and 11 exactly, we corrected for age (a) day 6: *n* = 19, day 7: *n* = 8; (b) day 10: *n* = 4, day 11: *n* = 26, day 12: *n* = 21); and (c) calculated growth rate as mass increase per day.

### Food provisioning observations

In total, we monitored the frequency of provisioning visits at 20 nests on day 10 of the nestling phase (2019/2020: *n* = 5; 2022/2023: *n* = 4, 2023/2024: *n* = 8, 2024/2025: *n* = 3). Cameras (GoPro Hero 6, GoPro, San Mateo, CA, powered by battery packs) were placed ∼2 m from the nest. Cameras were all placed before 07:00 h and collected around 16:00 h, resulting in an average duration of recording of almost 10 h (range = 5.94 to 12.28 h, *n* = 17; 2 cameras had stopped working within 2.5 h, whereas at 1 nest it was raining during the recording; we omitted these 3 recordings from the analyses). Birds resumed provisioning very soon after camera placement, and therefore the entire recording at each nest was used for quantifying food provisioning rates. Provisioning visits were quantified by watching videos, using the software BORIS, version 8.9.4 (Friard and Gamba 2016), which was used to timestamp provisioning behavior when playing videos.

### Statistical analyses

All analyses were conducted in RStudio using R (version 4.4.3; R Core Team 2025). Data visualization was performed using the *ggplot2* package (Wickham 2016). All linear mixed-effects (LMM) models were fitted using *lme4* (Bates et al. 2015), whereas we assessed normality and homoscedasticity by visually inspecting residual Q–Q plots and residuals-versus-fitted plots. Generalized linear mixed models (GLMMs) were fitted using either *lme4* or *glmmTMB* (Brooks et al. 2017), depending on the error distribution. For GLMMs with Poisson errors (and log link), we examined residual diagnostics and calculated Pearson dispersion parameters to check for overdispersion. We checked all independent variables for multicollinearity using variance inflation factors (VIFs) from the *car* package (Fox et al. 2019), and confirmed that VIF values remained below standard thresholds for concern (all VIF < 1.05).

To test whether females in larger groups laid larger clutches (ie more eggs per nest), we analyzed the complete clutch size of all nests found before, or during, the egg stage, excluding cuckoo eggs in our count of clutch size. Clutch size was modeled using a Poisson GLMM with group size as a fixed effect, and group identity as a random intercept to account for repeated measurements from specific groups.

To test whether larger groups produced more fledglings per breeding attempt, we fitted a negative binomial GLMM (due to a large number of nests producing no fledglings) with number of babbler fledglings in each nest as a response variable and group size as a predictor, with group identity as random intercept. All nests were included, and nests which produced no fledglings were assigned a fledgling count of zero. Because many nests failed during the egg stage, we repeated this analysis, including only nests in which at least 1 egg had hatched. To account for the fact that clutch size sets an upper limit on fledgling number, we also modeled egg-to-fledgling success as the proportion of eggs which produced a fledgling per nest (with the number of fledglings as “successes” and the number of eggs which did not produce fledglings as the number of “failures”) in a GLMM with beta-binomial error. This model included group size as a fixed effect and group identity as a random intercept. Excluding nests parasitized by Levaillant's cuckoos (see above) did not change the conclusions (Table S6).

To analyze the relationship between egg volume and group size, we fitted an LMM with individual egg volume as the response variable and group size and clutch size as fixed effects. Nest identity and group identity were included as a random intercepts to account for repeated measurements within nests and groups.

The effect of group size on babbler nestling mass was analyzed in 3 ways. First, we examined predictors of mass at day 6 using an LMM with mass of each nestling as the response variable, group size and actual nestling age (day 6 or 7, see Table 1) as fixed effects, and nest identity and group identity as a random intercepts. Second, we repeated this modeling approach for nestling mass around day 11 (see Table 1), using a larger dataset that also included nestlings from breeding seasons where only day 11 measurements were available. Third, we analyzed nestling growth rate between day 6 and day 11, calculated as the increase in body mass divided by the number of days between measurements. For this analysis, we fitted an LMM with growth rate of each nestling as the response, group size as a fixed effect, and nest identity and group identity as random intercepts. Because sample sizes were limited relative to the number of predictors for the analyses of nestling mass and growth rate, we did not include brood size (ie number of nestlings) in the initial models. However, doing so did not change the group size effects (Table S5) and therefore we report the results of models without brood size. We repeated these analyses excluding the 3 nests which contained a Levaillant cuckoo nestling, but this did not change the conclusions (Table S7).

We modeled provisioning as the number of feeds recorded during observation periods of varying duration using a negative binomial GLMM with a log link error. The number of provisioning visits (per nest) was the response variable, and group size and brood size were included as predictors. To account for variation in observation effort, we included an offset of log(observation hours), so that model coefficients described effects on the expected number of feeding visits per hour. Group identity was included as a random intercept. Repeating the analysis excluding the 3 broods that contained a Levaillant's cuckoo nestling did not change the results (Table S8).

Note that sample sizes across analyses differ, for example because clutch size and egg size were unknown if nests had been found after hatching, because eggs were predated or hatched before we could measure them, or because not all nestlings were measured on day 6.

## Results

**Figure 1:**
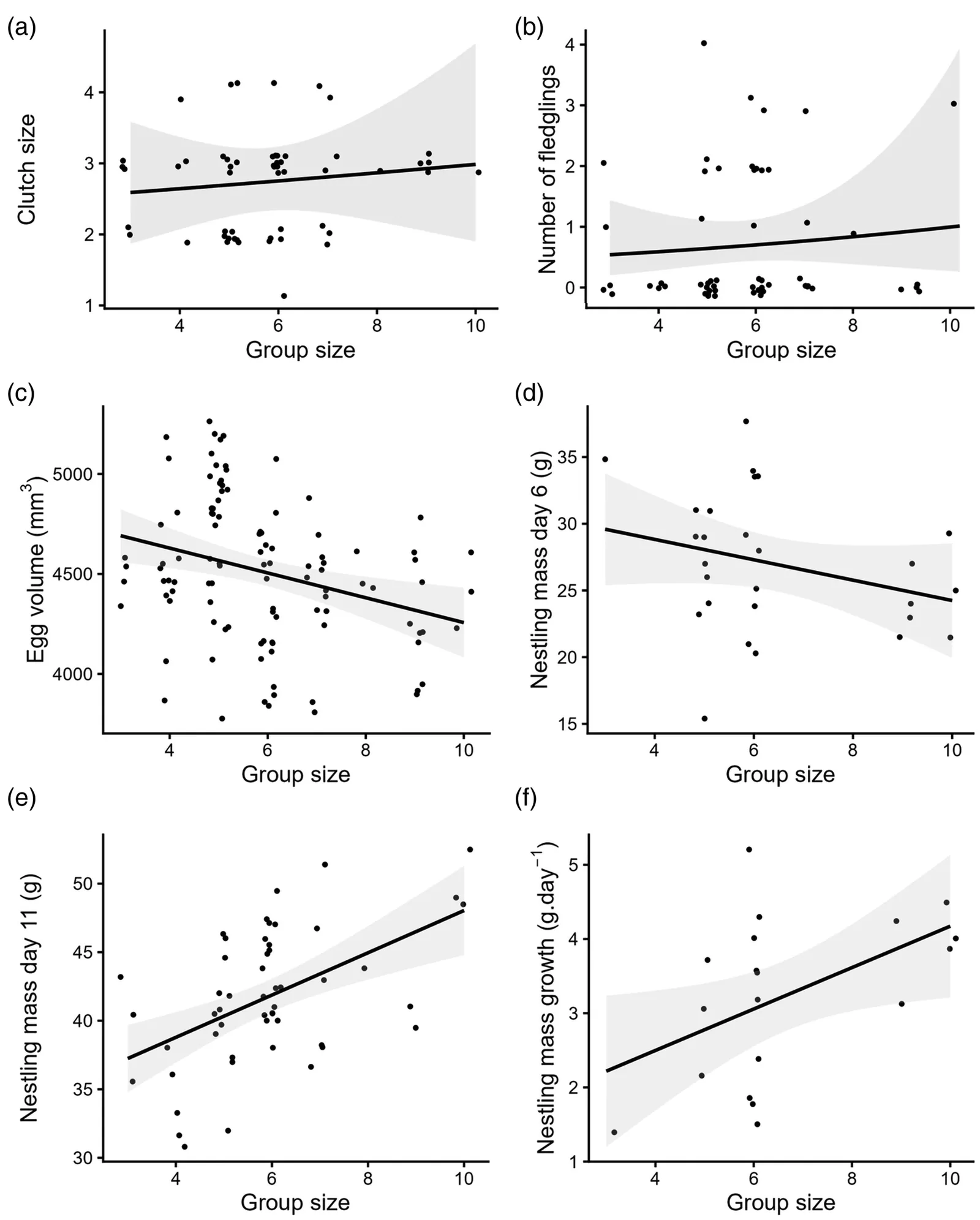
Relationships between group size and reproductive, egg and nestling measurements in arrow-marked babblers. We plotted, as a function of group size: a) clutch size (number of eggs; *n* = 54 nests for which final clutch size was known; *P* = 0.693), b) number of fledglings produced per nest (*n* = 56 nests; *P* = 0.090), c) egg volume (*n* = 107 eggs from 41 nests; *P* = 0.035), d) nestling mass (g) around day 6 (*n* = 27 nestlings from 13 nests; *P* = 0.195), e) nestling mass (g) around day 11 (*n* = 51 nestlings from 24 nests; *P* = 0.002), and f) nestling growth rate (mass increase in g day^−1^) between day 6 and day 11 (*n* = 19 nestlings from 9 nests; *P* = 0.050). Dots reflect individual nests (a) and (b), individual eggs (c), or individual nestlings (d–f). The solid lines and shaded bands reflect the model-predicted relationships and 95% confidence intervals, respectively.

Median complete clutch size of 54 nests which were found before hatching and for which clutch size was known was 3 eggs (mean: 2.741 ± 0.678 [SD] eggs; 1 egg: *n* = 1, 2: *n* = 18; 3: *n* = 29; 4: *n* = 6). Most nests (37/56, including those nests for which exact clutch size was unknown) did not produce any fledglings, but out of nests which produced fledglings, the median number of fledglings was 2 (overall mean: 0.70 ± 1.09 [SD] fledglings; zero fledglings: *n* = 37, 1: *n* = 5, 2: *n* = 9; 3: *n* = 4; 4: *n* = 1). Compared with smaller groups, larger groups of babblers did not lay larger clutches (β = 0.020 ± 0.051 SE, *z* = 0.395, *P* = 0.693; Fig. 1a). Larger groups also did not produce more fledglings, either when including all 56 nests (β = 0.090 ± 0.156 SE, *z* = 0.578, *P* = 0.564; Fig. 1b; Table S1b) or the 35 nests which hatched at least 1 egg (β = 0.059 ± 0.120 SE, *z* = 0.487, *P* = 0.626; Table S1c). Finally, the proportion of laid eggs that produced fledglings in each of the 54 nests (where clutch size was known) was also not related to group size (β = −0.005 ± 0.169 SE, *z* = −0.032, *P* = 0.975; Table S2).

Egg volume was highly variable (mean ± SD: 4503 ± 358 mm^3^, range: 3,777 to 5,263 mm^3^; *n* = 107 eggs from 41 nests). After correcting for clutch size, females in larger groups laid significantly smaller eggs (β = −76.243 ± 34.615 SE, *t* = −2.203, *P* = 0.035; Fig. 1c). Clutch size did not predict egg volume (β = −3.346 ± 51.026 SE, *t* = −0.066, *P* = 0.948; Table S3).

Group size did not predict nestling mass at day 6 (or 7), after accounting for the effect of nestling age (Table 1A, Fig. 1d). Around day 11, nestlings in larger groups were significantly heavier after accounting for repeated measurements within nests and social groups (Table 1, Fig. 1e), but at this stage mass was unrelated to the actual age of nestlings (Table 1B). Nestling growth rate was positively associated with group size, although this effect was not quite significant (*P* = 0.050; Table 1C, Fig. 1f). In the day 6 mass and growth-rate models, the random-effect variance for group identity was estimated as zero, indicating no detectable additional among-group variation after accounting for nest identity and fixed effects.

**Figure 2:**
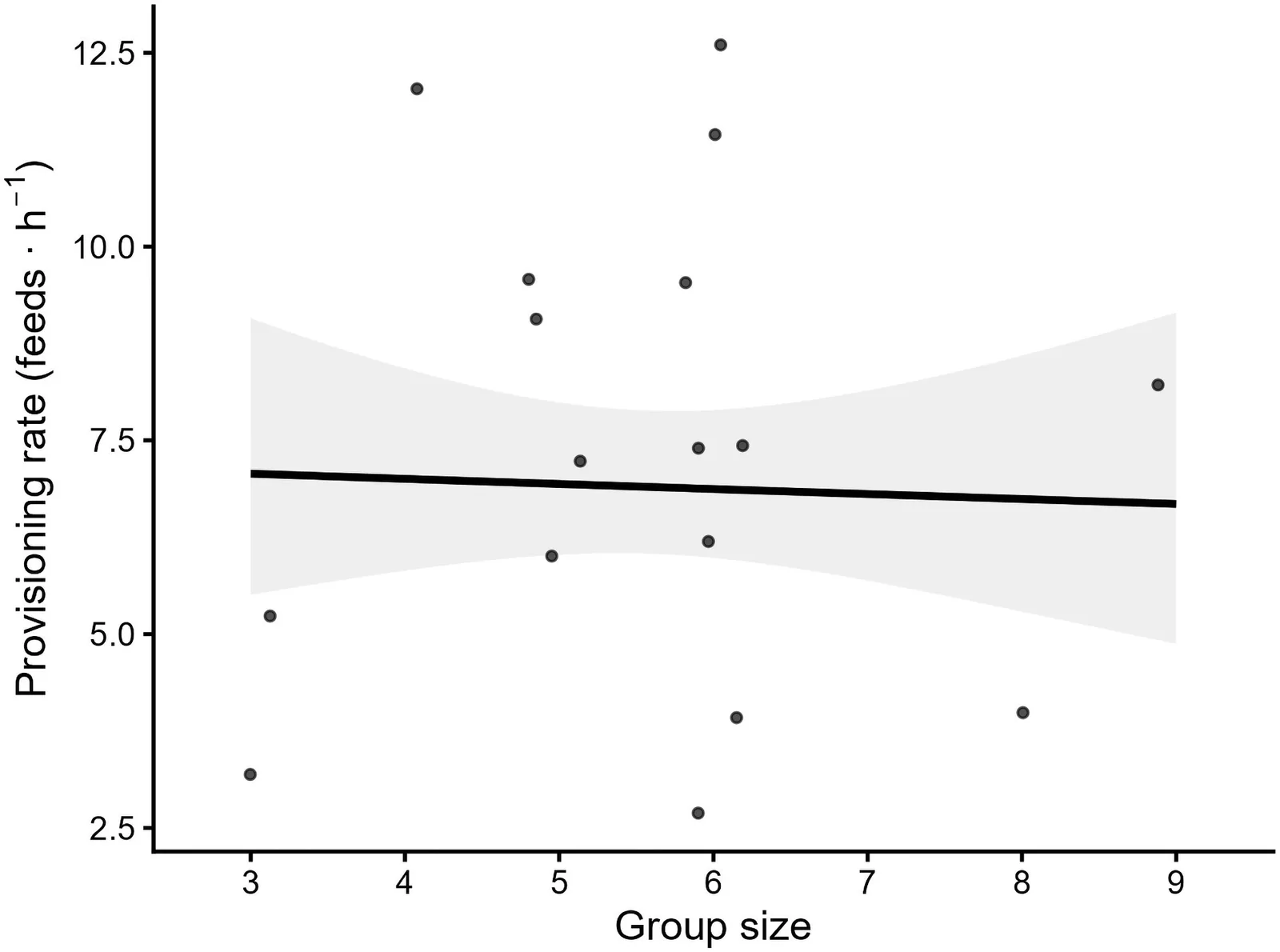
Relationship between provisioning rate and group size during the late nestling stage (day 10) in 17 arrow-marked babbler broods. Dots reflect observed provisioning rates per nest, averaged over the day's recording as the number of provisioning visits divided by number of hours observed. The solid line shows the model-predicted relationship from a negative binomial mixed model with brood size included as a covariate and group identity included as a random intercept (full model output is given in Table S5); the shaded band shows the 95% confidence interval.

Observed average provisioning rates varied substantially among nests, ranging from ∼2 to 4.5 food items per hour per nestling. However, provisioning rates were not related to group size (β = −0.009 ± 0.042 SE, z = −0.224, *P* = 0.823; Fig. 2; Table S5), after accounting for the fact that larger broods received more provisioning visits per hour (β = 0.459 ± 0.062 SE, z = 7.460, *P* < 0.001; Table S5).

## Discussion

Our results reveal that arrow-marked babbler females in larger groups laid smaller eggs, but that, just before fledging, their nestlings were heavier than those from smaller groups. These findings suggest that mothers reduced prenatal investment in the presence of more helpers, and that the developmental trajectory of offspring mass was subsequently overcompensated. Supporting this, nestlings across all groups were of equal size when 6 days old, but those in larger groups showed a tendency for faster mass gain in the subsequent days, consistent with compensatory growth in larger groups. Together, these findings suggest that helpers provide a dual benefit, both improving offspring quality and facilitating prenatal load-lightening of breeding females.

Our results of dual benefits illustrate that using the term “concealed-helper-effects” for “prenatal load-lightening” is potentially misleading, because we show that despite smaller eggs, fledglings can nonetheless be measurably larger. In the discussion below, we therefore use the term “prenatal load-lightening” instead to be more specific, and we encourage future work to do so also.

### Prenatal load-lightening

Across cooperative breeders, support for the prenatal load-lightening hypothesis is mixed, with some species showing reduced egg size when breeding with helpers and others showing no such pattern (Dixit et al. 2017). In our study, the combination of smaller eggs in larger groups and greater nestling mass by day 11 suggests that females reduce energetic investment at the egg stage when they may rely on group members to support later stages of development. Egg production is energetically and nutritionally costly (Monaghan and Nager 1997; Williams 2005) and reducing egg size could help breeders conserve resources for future reproduction (Arnold and Owens 1998; Russell et al. 2007). However, in general, load-lightening may be a particularly beneficial strategy in cooperatively breeding species where long lifespans and long breeding seasons favor future over immediate reproduction, as may well be the case in babblers (Arnold and Owens 1998; Covas and Griesser 2007). Arrow-marked babblers are long-lived, experience high nest predation (manuscript in prep.), and can re-nest repeatedly within a season, making reduced maternal prenatal investment a plausible strategy in this species (although whether the reduction in egg size indeed has a positive effect on females and on future reproductive success remains to be tested). A broader comparative analysis explicitly linking egg traits to nest failure regimes, pace of life, and helper effects would be needed to explain why some cooperative breeders exhibit these patterns, whereas others do not (see Dixit et al. 2017).

One complication of studies on cooperative breeders is that correlates of group size, such as egg size, might be the indirect result of other, correlated factors (Cockburn et al. 2008). For example, increased competition for food within larger groups might result in reduced egg size. In larger babbler groups, however, competition over food seems unrelated to group size because larger groups occupy larger territories with presumably more food (Monadjem et al. 1995; see also Downing and Helanterä 2026). Equally, aspects such as female age and experience, as well as tenure of breeders, may influence egg size and at the same time be associated with group dynamics, but unfortunately data on these aspects were not available for our study. Thus, the extent to which smaller eggs truly reflect reduced maternal investment (ie reduced costs), rather than an unmeasured correlate of group size, remains to be tested. Future work specifically needs to quantify female age, experience, and condition and survival in relation to group size and egg size, or to use experimental approaches such as the temporary removal of helpers or supplementing food to disentangle the effects of helper presence and environmental circumstances.

Beyond these correlational limitations, egg size in itself represents only one aspect of maternal investment in eggs and in determining offspring size and condition. Hatchling size and condition are also shaped by egg composition, as smaller eggs often produce smaller hatchlings (Williams 1994; Krist 2011), in part because they may contain lower levels of egg yolk and other nutrients (Williams 1994; Badzinski et al. 2002). Additionally, small eggs might contain reduced levels of other components, such as hormones, antibodies, or immune factors (Groothuis et al. 2005; Gil 2008). If so, nestling growth patterns could have been independent of egg size itself, but instead reflect lasting effects of these egg components on embryonic development, physiology, and stress responses of hatchlings (Groothuis et al. 2005; Gil 2008). Whether and how these factors vary with differing degrees of cooperative breeding and how they interact with post-hatching development remains poorly tested. Only a limited number of studies on cooperative breeders have explicitly linked group size or helper presence to egg composition and hormone profiles (Paquet et al. 2013; Fortuna et al. 2023). Work on sociable weavers illustrates these complex dynamics: in 1 study, females breeding with helpers laid smaller eggs with reduced yolk androgen and corticosterone levels (Paquet et al. 2013), whereas a subsequent long-term analysis found egg mass to be highly repeatable within females and unrelated to helper number (Fortuna et al. 2021). More recent work further demonstrated that specific egg components, including yolk mass and lipid content, can nonetheless vary positively with group size and laying order (Fortuna et al. 2023). Together, these studies highlight that helper effects on maternal allocation may act on egg composition as well as egg size, and similar analyses in arrow-marked babblers would be valuable to determine whether reduced egg size in larger groups is accompanied by systematic changes in egg composition, and how this, in turn, relates to hatchling and nestling condition.

### Compensatory offspring growth

We found that females in larger groups produced smaller eggs, resulting in nestlings that tended to be only slightly lighter at 6 days old, and although this should be interpreted with caution due to limited statistical power, the effect of group size was not significant. We did not measure mass of the nestlings immediately after hatching, but under the predictions of prenatal load-lightening, we expect hatchlings from larger groups to be initially lighter because eggs were smaller (as in Russell et al. 2007). If so, on day 6 of nestling age, this effect had largely disappeared. This suggests that compensatory growth and associated helper care have already begun to take effect. In line with this, we show that nestlings in larger groups grew faster (after day 6) and were heavier by day 11 than those from smaller groups. This pattern suggests a combination of prenatal maternal load-lightening and postnatal helper benefits which result in a net positive effect of helper presence on group reproductive success in terms of offspring quality.

Compensatory growth in offspring resulting from helper contributions has been demonstrated in some cooperatively breeding species, where reduced egg investment in larger groups is offset by increased postnatal care, such that fledgling mass does not differ between broods with many versus few helpers (Russell et al. 2007; Santos and Macedo 2011). In carrion crows, females breeding with more subordinate male helpers lay smaller eggs but nevertheless produce heavier nestlings, indicating that helpers not only compensate for initially smaller offspring but actually over-compensate to produce nestlings in better condition later in development (Canestrari et al. 2011). We find similar patterns in our population of arrow-marked babblers: nestlings from larger groups are heavier by day 11 than those from smaller groups. Having larger nestlings can provide various fitness benefits for groups. Cooperative breeders tend to have prolonged postfledgling dependence of offspring (Langen 2000; Russell 2001), a phase with high rates of mortality. The postfledging period is a particularly vulnerable period for young, especially in predator-rich environments (Naef-Daenzer and Grüebler 2016), and fledglings which are stronger and larger during this phase experience higher chances of survival (Mumme 1992; Monrós et al. 2002). Thus, a key benefit of cooperative breeding in arrow-marked babblers may be that large groups can produce fledglings that are in better condition with higher chances of survival, despite originating from small eggs, which in turn allows breeding females to optimize their future reproductive performance. Additionally, nestlings which are bigger may fledge earlier than smaller ones. Although not explicitly tested here, it could be that the larger nestlings observed in larger groups fledge earlier than those in smaller groups, reducing time spent in the vulnerable nestling stage. Postfledging survival and behavior are often not well documented due to the challenges associated with monitoring this stage (Thompson and Ridley 2013) and we argue that future work focusing on how fledgling condition and group size relate to survival and fitness could provide interesting insights into cooperative breeding in this and other species.

Interestingly, we found no evidence that provisioning rates increase with group size. We recorded each nest for only 1 day and did not correct for potential varying circumstances on the days of recording, but because provisioning rates to larger broods were higher, we believe that the pattern is representative at least for the later nestling stage when we recorded the nests (10-day-old nestlings). The fact that nestlings were nonetheless heavier at this stage suggests either that any compensation by helpers must have taken place earlier (specifically when nestlings were still smaller in larger groups) or that the mechanism underlying compensation is not simply higher feeding frequency itself; helpers might contribute to nestling condition through other forms of care. For example, additional helpers can increase brooding or sheltering, improving thermoregulation and reducing the energetic costs of maintaining body temperature for small nestlings, or allow breeders to allocate more time to vigilance and self-maintenance (Hatchwell 1999; Hatchwell and Komdeur 2000). Helpers may also coordinate or synchronize provisioning visits, which can reduce begging effort and disturbance at the nest, potentially lowering energy expenditure and stress (Raihani et al. 2010; Trapote et al. 2024). All of these mechanisms have the potential to result in larger, healthier offspring in larger babbler groups in the absence of a measurable increase in nestling provisioning rate. Understanding the relative importance of these different pathways of helper contribution in cooperative breeders is an important next step for quantifying the benefits of cooperative breeding in general, and in terms of understanding prenatal load-lightening in particular.

An alternative explanation for our results, and indeed for many existing reports of prenatal load-lightening, is that helper presence is confounded with other factors which affect maternal investment and postnatal offspring condition. For example, carers in larger groups may be able to invest more in food quality, eg by being able to spend more time searching for higher-quality food items and thus provide nestlings equally frequently but with food that is richer in nutrients. Across taxa, larger groups forage more efficiently (Beauchamp 1998), and previous work has shown that larger groups of babblers hold larger territories (Monadjem et al. 1995), both of which could improve access to richer resource patches. Another interesting possibility is that maternal deposition of important egg contents, such as antioxidants and hormones, varies with maternal quality, which is indirectly linked to group size. Different growth trajectories of smaller and larger nestlings as a result of differences in egg content may arise if females in better condition (or larger groups) traded off investment in egg size for higher quality egg content: eg increased hormonal investment in eggs may promote nestling development (Hinde et al. 2009). Providing evidence for causal effects of helper presence on prenatal load-lightening and postnatal additive helper benefits requires studies which can control for or experimentally exclude variation in territory and breeder quality; although this is difficult to achieve in wild systems, such studies have the potential to provide greater clarity on the mechanisms underlying group size-related variation in reproductive outcomes.

### Load-lightening and compensatory offspring growth: do offspring pay the price?

One aspect which has not been studied in cooperative breeders which exhibit prenatal load-lightening is whether compensatory growth incurs negative consequences for offspring at a later age. Increased metabolic load, oxidative stress, altered endocrinology, reduced lifespan and future reproductive success, as well as personality traits, have all been linked to compensatory growth in noncooperative species (Naguib and Gil 2005; Naguib et al. 2006; Monaghan 2008; Krause and Naguib 2011; Hector and Nakagawa 2012). If compensatory growth is associated with delayed costs for survival or performance (Metcalfe and Monaghan 2001; Fisher et al. 2006; Hector and Nakagawa 2012), the combination of reduced egg size and enhanced nestling mass in larger groups suggests a shift in the distribution of costs, with females potentially saving resources at the egg stage but offspring paying at least some of the price through compensatory growth. Our study did not measure physiological markers or long-term fitness, but the combination of reduced egg size and enhanced posthatching growth raises the possibility that offspring from larger groups may incur hidden costs that are not apparent from mass alone. However, a study on telomere length, a marker of somatic condition and predictor of long-term survival, in sociable weavers (*Philetairus socius*) has shown that helper presence actually improves telomere length of nestlings (Quque et al. 2021). This suggests that negative effects of compensatory growth are likely outweighed by benefits of additive helper care. In purple-crowned fairy wrens *Malurus coronatus*, nestling telomere length is unaffected by helper number apart from at the largest group sizes (3+ helpers), where density-dependent food competition appears to drive a reduction in nestling telomere length (Eastwood et al. 2022). An important next step will be to test whether nestlings from larger babbler groups differ in physiological condition, physiology, stress indicators, or later survival and recruitment, and to assess whether a broader range of cooperative breeders experience the same, reduced, or completely different trade-offs associated with compensatory growth compared with noncooperative species. Thus, although load-lightening and compensatory growth seem beneficial aspects of cooperative breeding in babblers, studies into the long-term effects are needed to determine whether this strategy has long-term costs for the offspring in this as well as other cooperatively breeding species.

### Conclusion

Our results contribute to a growing recognition that the benefits of cooperative breeding may be relatively “hidden” in subtle reductions of breeder investment (Dixit et al. 2017). Moreover, the results illustrate that when assessing the benefits of cooperative breeding it is important to investigate offspring development across stages and explore factors beyond provisioning rate and fledgling number: In arrow-marked babblers, larger groups produced smaller eggs, yet heavier nestlings just before fledging, consistent with the idea that helpers in larger groups can compensate for reduced prenatal investment in offspring (even though we did not find that food provisioning rates increase with group size). Interestingly, unlike many studies that support the “prenatal load-lightening” hypothesis, we demonstrate a dual benefit of helpers in this species as not only could breeders reduce investment, offspring were ultimately also in better condition. We argue that differences in life history, particularly determined by the relative importance of current versus future reproduction, may explain why the mechanisms by which helping is selected differ so vastly between species.

## Supplementary Material

arag095_Supplementary_Data

## Data Availability

Analyses reported in this article can be reproduced using the data provided by Janse van Vuuren et al. (2026).
