# Supplementary material for: Group size predicts prenatal investment and offspring growth in a cooperatively breeding bird
Source: Behav Ecol. 2026 Sep 3;37(5):arag095. doi: 10.1093/beheco/arag095 (PMC13540718; doi:10.1093/beheco/arag095)
Supplement: Supplementary file 1 [file arag095_supplementary_data.doc]

**Group size predicts pre-natal investment and offspring growth in a cooperatively breeding bird**

**Supplementary material**

**Table S1. Effects of group size on clutch size and fledgling number in arrow-marked babblers**. Clutch size was analyzed using a Poisson mixed model, while fledgling number was analyzed using negative binomial mixed models. All models included group identity as a random intercept.

|  | **(A) Clutch size**  **(n = 54 nests, 25 groups)** | | |  | **(B) Fledgling number: all nests (n = 56 nests, 26 groups)** | | |  | **(C) Fledgling number: hatched nests (n = 35 nests, 20 groups)** | | |
| --- | --- | --- | --- | --- | --- | --- | --- | --- | --- | --- | --- |
|  | **Estimate ± SE** | ***z*** | ***P*** |  | **Estimate ± SE** | ***z*** | ***P*** |  | **Estimate ± SE** | ***z*** | ***P*** |
| Intercept | 0.891 ± 0.310 |  |  |  | -0.887 ± 0.938 |  |  |  | -0.236 ± 0.736 |  |  |
| Group size | 0.020 ± 0.051 | 0.395 | 0.693 |  | 0.090 ± 0.156 | 0.578 | 0.564 |  | 0.059 ± 0.120 | 0.487 | 0.626 |
|  |  |  |  |  |  |  |  |  |  |  |  |
| ***Random intercept*** | **Variance ± SD** |  |  |  | **Variance ± SD** |  |  |  | **Variance ± SD** |  |  |
| Group identity | <0.001 ± <0.001 |  |  |  | <0.001 ± <0.001 |  |  |  | <<0.001 ± <0.001 |  |  |

**Table S2. Effect of group size on the likelihood that an egg produced a fledgling in arrow-marked babblers.** Fledging success was analyzed as the number of eggs that produced fledglings using a beta-binomial mixed model with clutch size as the denominator. Group identity was included as a random intercept.

|  | **Egg-to-fledgling success**  **(n = 54 nests, 25 groups)** | | |
| --- | --- | --- | --- |
|  | **Estimate ± SE** | ***z*** | ***P*** |
| Intercept | −1.294 ± 1.032 |  |  |
| Group size | 0.044 ± 0.171 | 0.258 | 0.796 |
| ***Random intercept*** | **Variance ± SD** |  | |
| Group identity | <0.001 ± <0.001 |  |  |

**Table S3. Effects of group size and clutch size on egg volume in arrow-marked babblers.** Egg volume was analyzed using a linear mixed-effects model with nest identity and group identity included as random intercepts.

|  | **Egg volume**  **(n = 107 eggs, 41 nests, 22 groups)** | | |  |  |
| --- | --- | --- | --- | --- | --- |
|  | **Estimate ± SE** | ***t*** | ***P*** | | |
| Intercept | 4979.216 ± 250.615 |  |  | | |
| Group size | −76.243 ± 34.615 | −2.203 | 0.035 | | |
| Clutch size | −3.346 ± 51.026 | −0.066 | 0.948 | | |
| ***Random intercepts*** | **Variance ± SD** |  |  | | |
| Nest identity | 34889 ± 186.8 |  |  | | |

**Table S4. Effects of group size and brood size on provisioning rate in arrow-marked babblers.** Provisioning was analyzed as the number of feeding visits using a negative binomial mixed model with a log link. Observation duration was included as an offset, and group identity was included as a random intercept.

|  | **Provisioning rate (n = 17 nests, 13 groups)** | | |  |
| --- | --- | --- | --- | --- |
|  | **Estimate ± SE** | ***z*** | ***P*** |  |
| Intercept | 0.957 ± 0.297 |  |  |  |
| Group size | -0.009 ± 0.042 | -0.224 | 0.823 |  |
| Brood size | 0.459 ± 0.062 | 7.460 | **<0.001** |  |
| ***Random intercept*** | **Variance ± SD** |  |  |  |
| Group identity | 0.036 ± 0.190 |  |  |  |

**Table S5.** **Effects of group size and brood size on nestling mass and growth in arrow-marked babblers.** Linear mixed-effects models tested effects of group size on (A) nestling mass around day 6, (B) nestling mass around day 11, and (C) growth rate between these stages. Age was included as a covariate in the mass models, and brood size was included as a covariate in all models. Nest identity and group identity were included as random intercepts.

|  | **(A) Nestling mass day 6**  **(n = 27 nestlings, 13 nests, 11 groups)** | | | **(B) Nestling mass day 11**  **(n = 51 nestlings, 24 nests, 17 groups)** | | | **(C) Nestling growth rate**  **(n = 19 nestlings, 9 nests, 8 groups)** | | | | |  |
| --- | --- | --- | --- | --- | --- | --- | --- | --- | --- | --- | --- | --- |
|  | **Estimate ± SE** | ***t*** | ***P*** | **Estimate ± SE** | ***t*** | ***P*** | **Estimate ± SE** | ***t*** | | ***P*** | |  |
| Intercept | 10.035 ± 14.453 |  |  | 41.593 ± 13.968 |  |  | 0.767 ± 1.120 |  | |  | |  |
| Group size | −0.235 ± 0.622 | −0.378 | 0.709 | 1.580 ± 0.466 | 3.390 | 0.003 | 0.198 ± 0.170 | 1.163 | | 0.294 | |  |
| Age | 3.695 ± 2.085 | 1.772 | 0.090 | −0.731 ± 1.303 | −0.561 | 0.580 |  |  | |  | |  |
| Brood size | −1.809 ± 1.684 | −1.075 | 0.294 | −0.354 ± 1.160 | −0.306 | 0.763 | 0.437 ± 0.547 | 0.798 | | 0.449 | |  |
|  |  |  |  |  |  |  |  |  | |  | |  |
| ***Random intercept*** | **Variance ± SD** |  |  | **Variance ± SD** |  |  | **Variance ± SD** |  | |  | |  |
| Nest ID | <0.001 ± <0.001 |  |  | 5.106 ± 2.260 |  |  | 0.122 ± 0.349 |  | |  | |  |
| Group identity | <0.001 ± <0.001 |  |  | 1.547 ± 1.244 |  |  | <0.001 ± <0.001 | |  | |  | |

**Table S6. Effects of group size on the number of arrow-marked babbler fledglings produced per nest after excluding nests parasitized by Levaillant’s cuckoos.** All-nest fledgling counts were analyzed using a negative binomial mixed model, while the hatched-nests-only analysis was analyzed using a Poisson mixed model. Group identity was included as a random intercept in both models.

|  | **All nests without cuckoos (n = 38 nests, 21 groups)** | |  | |  | **Hatched nests without cuckoos (n = 23 nests, 16 groups)** | |  | |  |  |
| --- | --- | --- | --- | --- | --- | --- | --- | --- | --- | --- | --- |
|  | **Estimate ± SE** | ***z*** | | ***P*** | | **Estimate ± SE** | ***z*** | | ***P*** | | |
| Intercept | −1.157 ± 1.201 |  | |  | | −0.639 ± 0.887 |  | |  | | |
| Group size | 0.139 ± 0.208 | 0.665 | | 0.506 | | 0.135 ± 0.148 | 0.909 | | 0.363 | | |
|  |  |  | |  | |  |  | |  | | |
| ***Random intercept*** | **Variance ± SD** |  | |  | | **Variance ± SD** |  | |  | | |
| Group identity | <0.001 ± <0.001 |  | |  | | 0.014 ± 0.118 |  | |  | | |
|  |  | |  | |  |  |  | |  | | |

**Table S7. Analyses of group size effects on arrow-marked babbler nestling mass and growth, excluding cuckoo parasitized nests.** Linear mixed-effects models tested whether the effects of group size on nestling mass and growth were robust after excluding nests parasitized by Levaillant’s cuckoos. Nest identity and group identity were included as random intercepts.

|  | **(A) Nestling mass day 6**  **(n = 22 nestlings,**  **10 nests, 8 groups)** | | | **(B) Nestling mass day 11**  **(n = 47 nestlings,**  **22 nests, 16 groups)** | | | | | **(C) Nestling growth rate**  **(n = 14 nestlings,**  **6 nests, 5 groups)** | | | | | |  |
| --- | --- | --- | --- | --- | --- | --- | --- | --- | --- | --- | --- | --- | --- | --- | --- |
|  | **Estimate ± SE** | ***t*** | ***P*** | **Estimate ± SE** | ***t*** | | ***P*** | | **Estimate ± SE** | | ***t*** | | ***P*** | |  |
| Intercept | -8.079 ± 17.407 |  |  | 43.063 ± 12.116 |  | |  | | 1.102 ± 1.034 | |  | |  | |  |
| Group size | -0.337 ± 0.560 | -0.601 | 0.555 | 1.878 ± 0.452 | 4.156 | | <0.001 | | 0.320 ± 0.158 | | 2.021 | | 0.107 | |  |
| Age | 5.956 ± 2.558 | 2.328 | 0.031 | -1.075 ± 1.057 | -1.017 | | 0.319 | |  | |  | |  | |  |
|  |  |  |  |  |  | |  | |  | |  | |  | |  |
| ***Random intercepts*** | **Variance ± SD** |  |  | **Variance ± SD** |  | |  | | **Variance ± SD** | |  | |  | |  |
| Nest identity | <0.001 ± <0.001 |  |  | 4.374 ± 2.091 |  | |  | | 0.427 ± 0.654 | |  | |  | |  |
| Group identity | <0.001 ± <0.001 |  |  | <0.001 ± <0.001 | |  | |  | | <0.001 ± <0.001 | |  | |  | |

**Table S8. Effects of group size and brood size on provisioning rate in arrow-marked babblers excluding broods containing Levaillant’s** **cuckoo nestlings.** Provisioning was analyzed as the number of feeding visits using a negative binomial mixed model with a log link. Observation duration was included as an offset, and group identity was included as a random intercept.

|  | **Provisioning rate excluding broods with cuckoo nestling**  **(n = 14 broods, 11 groups)** | | |  |
| --- | --- | --- | --- | --- |
|  | **Estimate ± SE** | ***z*** | ***P*** |  |
| Intercept | 0.862 ± 0.420 |  |  |  |
| Group size | 0.007 ± 0.063 | 0.111 | 0.912 |  |
| Brood size | 0.465 ± 0.070 | 6.606 | <0.001 |  |
| ***Random intercept*** | **Variance ± SD** |  |  |  |
| Group identity | 0.045 ± 0.211 |  |  |  |
